# Rapid Inpatient Uptitration of Inhaled Treprostinil in PH-ILD Patients with Severe Phenotype

**DOI:** 10.3390/arm94010007

**Published:** 2026-01-09

**Authors:** Chebly Dagher, Allysse Thomas, Suzie Al Absi, Brett Carollo, Garrett Fiscus, Raj Parikh

**Affiliations:** 1 Department of Internal Medicine, University of Connecticut, Farmington, CT 06032, USA; 2 Department of Osteopathic Medicine, A.T. Still University School of Osteopathic Medicine-Arizona, Mesa, AZ 85206, USA; 3 Division of Pulmonary, Critical Care and Sleep, Hartford Hospital, Hartford, CT 06106, USA

**Keywords:** pulmonary hypertension, interstitial lung disease, inhaled treprostinil, rapid uptitration, prostacyclin therapy, severe PH-ILD, hemodynamics, inpatient protocol, pulmonary vascular resistance, right heart catheterization

## Abstract

**Highlights:**

**What are the main findings?**

- Rapid inpatient uptitration of inhaled treprostinil allowed all six severe PH-ILD patients to reach therapeutic dosing within one week.
- The protocol was well tolerated and associated with early hemodynamic improvement without dose interruptions or 90-day readmissions.

**What are the implications of the main findings?**

- Accelerated inpatient uptitration may offer a safe and practical strategy for high-risk PH-ILD patients who require faster therapeutic optimization.
- This approach provides a protocol for future prospective studies evaluating early, aggressive prostacyclin escalation in severe PH-ILD.

**Abstract:**

Pulmonary hypertension associated with interstitial lung disease (PH-ILD) is a progressive condition with limited treatment options and associated with high mortality rates. Inhaled treprostinil (iTre) is the only approved therapy for PH-ILD and has been shown to improve exercise capacity and delay disease progression. However, the conventional outpatient titration schedule requires 8–16 weeks to achieve therapeutic dosing, which may delay clinical benefit in those with advanced disease. We conducted a retrospective study of six patients with severe PH-ILD admitted to a tertiary academic center for initiation of iTre using a rapid inpatient uptitration protocol. iTre was started at 3 breaths four times daily (QID) and increased by 2 additional breaths every 12–24 h as tolerated, aiming for ≥9–12 breaths QID within one week under close monitoring. All six patients achieved target dosing without dose reduction or interruption. At three-month follow-up, mean pulmonary artery pressure decreased from 42 ± 5.5 to 35.2 ± 4.5 mmHg, pulmonary vascular resistance from 8.0 ± 1.2 to 6.0 ± 0.9 WU, and cardiac index increased from 2.05 ± 0.13 to 2.15 ± 0.12 L/min/m^2^. No readmissions occurred within 90 days. This study demonstrates that rapid inpatient uptitration of iTre in severe PH-ILD is feasible and well-tolerated, with preliminary evidence of short-term hemodynamic improvement.

## 1. Introduction

Pulmonary hypertension associated with interstitial lung disease (PH-ILD) represents a complex and high-risk clinical entity characterized by progressive functional decline, increasing oxygen requirements, and poor overall survival [1,2,3]. Patients who develop severe PH-ILD demonstrate more advanced hemodynamic impairment and worse outcomes, emphasizing the importance of timely recognition and effective therapeutic intervention [4,5].

Historically, severe PH-ILD has been defined by a mean pulmonary artery pressure (mPAP) ≥ 35 mmHg or an mPAP ≥ 25 mmHg with a cardiac index < 2.0 L/min/m^2^ [6,7]. More recently, the European Society of Cardiology (ESC) and European Respiratory Society (ERS) guidelines have proposed a refined hemodynamic definition, identifying severe disease as pulmonary vascular resistance (PVR) > 5 Wood units (WU) [8,9,10]. At present, inhaled treprostinil (iTre) remains the only therapy approved for the treatment of PH-ILD, based on the pivotal INCREASE trial [1,11]. In a subsequent post-hoc analysis, a dose–response relationship was observed, showing that patients achieving higher iTre doses experienced greater improvements in exercise capacity and reductions in disease progression [11,12]. These findings suggest that timely achievement of therapeutic dosing is clinically meaningful.

However, the conventional outpatient uptitration schedule for iTre is intentionally gradual, requiring 8 to 16 weeks to reach the therapeutic target of at least 9 breaths per treatment session, to allow patients to acclimate to common side effects [9,11]. While appropriate for most patients, this prolonged titration may delay potential benefits for those with severe PH-ILD, especially individuals exhibiting progressive symptoms or evidence of right ventricular (RV) dysfunction, in whom even short delays in optimizing pulmonary vasodilation can contribute to further decompensation [7].

Recognizing this limitation, we developed and implemented a novel, multidisciplinary inpatient rapid-uptitration protocol for iTre. The protocol was designed to safely achieve therapeutic dosing within one week under close hemodynamic and clinical monitoring. In this case series, we describe the feasibility, safety, and short-term hemodynamic outcomes of this approach in patients with severe PH-ILD, providing a clinical pathway for early, aggressive management of this high-risk phenotype.

## 2. Materials and Methods

We performed a retrospective analysis of six consecutive patients with severe PH-ILD admitted between January 2021 and January 2024. The diagnosis of ILD was confirmed by computed tomography (CT) showing diffuse parenchymal lung disease. Pre-capillary PH was confirmed by right heart catheterization (RHC), mPAP ≥ 20 mmHg, PCWP ≤ 15 mmHg, and PVR > 3 WU. Other causes of pre-capillary PH, including chronic thromboembolic pulmonary hypertension (CTEPH) and connective tissue disease, were excluded. Severe PH-ILD was defined as mPAP ≥ 35 mmHg, or mPAP ≥ 25 mmHg with a cardiac index (CI) < 2.0 L/min/m^2^, or by the more recent definition of PVR > 5 WU established by the 2022 European Society of Cardiology/European Respiratory Society (ESC/ERS) guidelines.

Patients were selected for inpatient rapid uptitration if they met criteria for severe PH-ILD, defined by invasive hemodynamics and in conjunction with high-risk clinical features such as worsening symptoms, RV dysfunction, or concern for clinical decompensation where delayed outpatient titration was felt to be suboptimal. Patients without these features were managed with standard initiation and titration.

There were no predefined exclusion criteria based on age, frailty, or comorbidities. Elderly patients and those with renal or hepatic comorbidities were not excluded a priori; eligibility for inpatient rapid uptitration was determined by the multidisciplinary PH team, and patients with severe, unstable non-cardiopulmonary illness or contraindications to prostacyclin therapy were not considered candidates.

**Figure 1:**
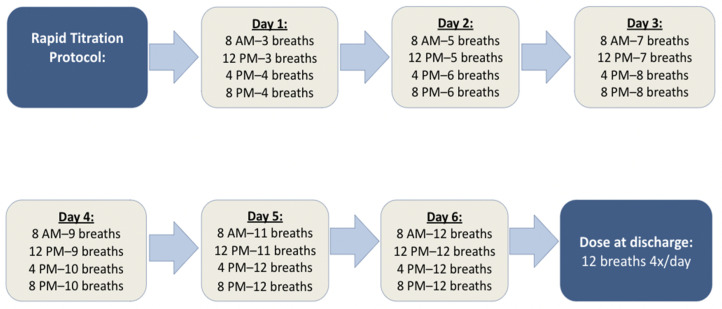
Rapid Inpatient Uptitration Protocol for Inhaled Treprostinil in Severe PH-ILD.

Patients who met these criteria were admitted for initiation and rapid titration of iTre at a single tertiary academic center under the guidance of the PH team. A rapid inpatient uptitration protocol, developed by our multidisciplinary PH team, was implemented with the goal of achieving therapeutic dosing (≥9–12 breaths QID) within one week while ensuring patient safety and tolerability. iTre was initiated at 3 breaths QID, with uptitration by 2 additional breaths per session every 12–24 h as tolerated, aiming for ≥9–12 breaths QID by hospital day 6 (Figure 1).

Dose escalation was individualized according to each patient’s clinical response, oxygenation, and symptom tolerance. Dose increases were deferred for predefined adverse-event criteria (detailed below), with re-initiation after symptom resolution. Supportive measures including pre-dose bronchodilator administration, acetaminophen or antiemetic therapy, and respiratory therapist-led (RT-led) technique optimization were used on an as-needed (PRN) basis to mitigate cough, headache, or nausea during uptitration.

Vital signs and oxygen saturation (SpO_2_) were assessed before and 30–60 min after each dose increase. Temporary dose holds or reductions were implemented in the event of symptomatic hypotension (systolic blood pressure (BP) < 90 mmHg or mean arterial pressure (MAP) < 60 mmHg), syncope/presyncope, sustained SpO_2_ < 88% or ≥4% decline from baseline, bronchospasm unresponsive to bronchodilator, or severe cough limiting therapy. Patients were discharged once stable on the target or highest tolerated dose for ≥24 h, with outpatient follow-up arranged. Adverse events (AEs) and tolerability were systematically documented throughout inpatient uptitration and follow-up. A dose-limiting AE was defined as any side effect preventing further titration for ≥24 h or requiring dose reduction.

Baseline hemodynamic parameters were obtained from the diagnostic RHC, and follow-up hemodynamics were reassessed after approximately 16 weeks of therapy to evaluate treatment response.

Paired comparisons between baseline and 3-month hemodynamic parameters were performed using paired-samples *t*-tests or Wilcoxon signed-rank tests as appropriate, with *p* < 0.05 considered statistically significant.

## 3. Results

The mean age of the cohort was 66 ± 7 years (IQR 62–71), with an equal sex distribution (3 males, 3 females). Three patients had idiopathic pulmonary fibrosis (IPF), two had combined pulmonary fibrosis and emphysema (CPFE), and one had nonspecific interstitial pneumonia (NSIP). Two patients were receiving antifibrotic therapy. Four of six (67%) required chronic supplemental oxygen at the time of PH diagnosis. The median duration of ILD prior to PH diagnosis was 5.5 months (IQR 4–6 months), and three patients (50%) had a history of smoking. The mean forced vital capacity (FVC) was 55.7 ± 3.7% predicted, and the mean diffusing capacity for carbon monoxide (DLCO) was 27.5 ± 8.8% predicted (Table 1).

At initial RHC (Table 2), the median right atrial pressure (RAP) was 6 mmHg (IQR 5–9), mPAP 42 mmHg (IQR 37–44), PVR 8.1 WU (IQR 6.6–8.5), and Fick-derived CI 2.1 L/min/m^2^ (IQR 2.0–2.2). All patients fulfilled hemodynamic criteria for severe pre-capillary PH (mPAP ≥ 35 mmHg or PVR > 5 WU).

All six patients (100%) achieved ≥9 breaths QID within 5 days (median 4 days, IQR 4–5). The mean discharge dose was 10.5 ± 2.3 breaths QID (Table 3). No patient required dose reduction or interruption. The titration process was performed under RT-led supervision with daily PH team evaluation.

All patients were able to complete the inpatient uptitration protocol and reached the target dose. Most experienced some degree of side effects, the most common being cough (4/6, 67%), followed by headache (2/6, 33%), dizziness (1/6, 17%), and nausea (1/6, 17%). These symptoms were generally mild to moderate, occurred early during uptitration, and were managed symptomatically with supportive measures such as bronchodilators or acetaminophen. No patient required dose reduction, interruption, or discontinuation of therapy. No readmissions were observed within 90 days of discharge.

Follow-up RHC after approximately 3 months of maintenance therapy demonstrated consistent hemodynamic improvement (Table 3). Compared with baseline, the mean mPAP decreased from 42 ± 5.5 to 35.2 ± 4.5 mmHg (Δ −6.8 mmHg, 16% reduction), and mean PVR decreased from 8.0 ± 1.2 to 6.0 ± 0.9 WU (Δ −2.0 WU, 25% reduction). The Fick CI modestly increased from 2.05 ± 0.13 to 2.15 ± 0.12 L/min/m^2^ (Δ +0.10 L/min/m^2^, 5% increase) (Table 4). Paired analysis demonstrated statistically significant reductions in mPAP and PVR and a modest but significant increase in cardiac index. No patient required escalation to additional pulmonary vasodilator therapy during the follow-up period.

## 4. Discussion

The INCREASE randomized trial established iTre as the first evidence-based therapy for PH-ILD, improving 6-min walk distance and delaying clinical worsening versus placebo [11]. Post-hoc analyses further suggested a dose–response relationship, with higher achieved iTre doses associated with greater physiologic benefit, including improvements in FVC [12]. These data imply that time to therapeutic dose may matter clinically, yet standard outpatient titration often requires 8–16 weeks to reach ≥9 breaths QID [11,13].

Our series addresses this gap by demonstrating the feasibility of a rapid inpatient uptitration strategy that achieved therapeutic dosing within one week under multidisciplinary monitoring, without interruptions, serious adverse events, or early readmissions, and with short-term hemodynamic improvement. In contrast to the conventional outpatient approach, inpatient titration allows continuous evaluation of symptoms, vital signs, and oxygenation, enabling real-time adjustments and proactive management of expected side effects such as cough, headache, or dizziness. The presence of a RT-led team and daily PH rounds provides an additional layer of safety that facilitates patient tolerance and adherence to the rapid uptitration protocol. Although dose escalation was individualized, it followed a structured inpatient protocol with predefined safety criteria.

This protocol did not require 1:1 nursing or dedicated RTs beyond standard inpatient staffing, with iTre administration incorporated into routine RT workflows and daily PH team rounds. However, inpatient initiation entails greater short-term resource utilization than outpatient titration and may be best suited to centers with established PH infrastructure.

Standard outpatient titration schedules are intentionally conservative and are primarily designed to allow gradual accommodation to prostacyclin-related symptoms rather than to mitigate established physiologic toxicity. Unlike parenteral prostacyclins, iTre has limited systemic exposure and no well-established risk of rebound pulmonary hypertension with dose escalation. In this context, close inpatient monitoring and incremental dose increases likely explain the favorable tolerability observed in this cohort.

Although prior reports have discussed practical management of inhaled prostacyclins (including mitigation of cough and bronchospasm) and device-specific considerations, none have specifically evaluated a structured, accelerated inpatient titration pathway in severe PH-ILD [9]. Our findings therefore complement INCREASE by focusing on a high-risk phenotype (severe disease) that may benefit from earlier attainment of effective prostacyclin exposure. Importantly, by 16 weeks of follow-up, our cohort demonstrated sustained hemodynamic improvements, with mPAP decreasing from 42 ± 5.5 to 35.2 ± 4.5 mmHg and PVR from 8.0 ± 1.2 to 6.0 ± 0.9 WU, accompanied by a modest increase in CI from 2.05 ± 0.13 to 2.15 ± 0.12 L/min/m^2^.

Although PVR decreased substantially, the associated increase in CI was modest. This likely reflects the complex pathophysiology of PH-ILD, in which improvements in pulmonary afterload do not always translate into proportional increases in cardiac output due to factors such as RV dysfunction, limited preload reserve, chronic hypoxemia, restrictive lung mechanics, and potential concomitant left ventricular diastolic dysfunction. In this context, stabilization or modest improvement in CI may still represent a clinically meaningful response, particularly over a short follow-up period.

This approach also fits a broader therapeutic context in Group 3 PH, where non-prostacyclin agents have yielded mixed or negative results. Endothelin-receptor antagonism with ambrisentan in IPF increased disease progression and respiratory hospitalizations (ARTEMIS-IPF) and was stopped early [14]. Riociguat in PH due to idiopathic interstitial pneumonias (RISE-IIP) increased serious adverse events and mortality, leading to early termination and regulatory warnings against its use in this population [15]. Trials of sildenafil have been largely negative on primary endpoints in advanced IPF, with benefits limited to selected secondary outcomes or subgroups (e.g., RV dysfunction) [16]. Against this backdrop, inhaled prostacyclin, targeting better-ventilated regions and theoretically minimizing V/Q mismatch, has a plausible mechanistic advantage that supports efforts to optimize dosing efficiently [9]. In addition, real-world experience suggests higher maintenance iTre doses can be tolerated in carefully monitored settings, aligning with our inpatient pathway’s goals [17].

This study has several limitations. First, it represents a single-center retrospective case series with a small sample size, which limits the generalizability of the findings. Second, the follow-up period was short (three months), preventing assessment of longer-term outcomes such as sustained hemodynamic improvement, functional capacity, or survival. Third, the absence of a control or comparison group precludes definitive conclusions about the causal relationship between rapid inpatient uptitration and the observed improvements. Additionally, while all patients tolerated the protocol well, this feasibility may reflect careful inpatient selection and multidisciplinary oversight, which may not be reproducible in all practice settings. Despite these limitations, the study provides important preliminary evidence supporting the safety and practicality of accelerated inpatient uptitration of iTre in severe PH-ILD and establishes a protocol for future prospective validation.

## 5. Conclusions

This study introduces a novel approach to the management of severe PH-ILD through rapid inpatient uptitration of iTre. Achieving therapeutic dosing within one week, compared with the conventional 8 to 16 weeks required in the outpatient setting, proved feasible and well-tolerated, providing preliminary evidence of short-term safety under close multidisciplinary supervision. Early hemodynamic improvement was observed without major adverse events or readmissions. This structured inpatient protocol offers a practical alternative for patients with advanced disease who may benefit from faster therapeutic optimization. Larger, prospective multicenter studies are warranted to validate these findings, assess long-term outcomes, and more definitively evaluate safety and rare adverse events.

## Figures and Tables

**Table 1:** Baseline demographic and clinical characteristics.

Patient ID	Age	Ethnicity	Sex	Prior Smoker	ILD Type	Length of ILD dx (Months)	Antifibrotic tx	FVC (%)	DLCO (%)	Use of Supplemental Oxygen
1	70	W	F	Y	CPFE	6	No	55	22	Yes
2	55	W	F	N	IPF	3	Yes	60	38	No
3	62	W	M	N	NSIP	5	No	59	39	No
4	75	W	F	N	IPF	5	No	54	24	Yes
5	71	AA	M	Y	CPFE	8	No	50	19	Yes
6	64	W	M	N	IPF	4	Yes	56	23	Yes

Baseline demographic and clinical characteristics of six patients diagnosed with pulmonary hypertension due to interstitial lung disease (PH-ILD). Abbreviations: W = White; AA = African American; F = Female; M = Male; Y = Yes; N = No; CPFE = Combined Pulmonary Fibrosis and Emphysema; IPF = Idiopathic Pulmonary Fibrosis; NSIP = Nonspecific Interstitial Pneumonia; FVC = Forced Vital Capacity; DLCO = Diffusing Capacity for Carbon Monoxide.

**Table 2:** Baseline Right Heart Catheterization Data at PH-ILD Diagnosis.

Patient ID	RAP (mmHg)	mPAP (mmHg)	PAWP (mmHg)	PVR (WU)	Fick CI
1	10	51	12	10.1	1.8
2	6	36	8	6.5	2.1
3	5	37	8	6.6	2.2
4	6	44	9	8.3	2.1
5	9	44	12	8.0	2.0
6	5	40	6	8.5	2.1

Invasive hemodynamic measurements obtained at baseline, prior to initiation of inhaled treprostinil therapy. Values represent resting right heart catheterization data for each patient. Abbreviations: RAP = Right Atrial Pressure; mPAP = Mean Pulmonary Artery Pressure; PAWP = Pulmonary Artery Wedge Pressure; PVR = Pulmonary Vascular Resistance; WU = Wood Units; CI = Cardiac Index.

**Table 3:** Hemodynamic Status Three Months After Discharge.

Patient ID	Inhaled Treprostinil Dose (Breaths QID)	RAP (mmHg)	mPAP (mmHg)	PAWP (mmHg)	PVR (WU)	Fick CI
1	18	8	42	10	7.3	2.0
2	15	6	30	7	5.0	2.3
3	12	6	31	10	4.6	2.3
4	12	5	34	7	6.1	2.2
5	12	8	38	13	6.3	2.0
6	12	8	36	8	7.0	2.1

Follow-up right heart catheterization data obtained approximately three months after hospital discharge, while on maintenance inhaled treprostinil therapy. Abbreviations: RAP = Right Atrial Pressure; mPAP = Mean Pulmonary Artery Pressure; PAWP = Pulmonary Artery Wedge Pressure; PVR = Pulmonary Vascular Resistance; WU = Wood Units; CI = Cardiac Index; QID = Four times daily.

**Table 4:** Hemodynamic Changes Following Three Months of Inhaled Treprostinil Therapy.

Parameter	Baseline (Mean ± SD)	3 Months (Mean ± SD)	Δ (Mean Change)	*p*-Value
mPAP (mmHg)	42 ± 5.5	35.2 ± 4.5	−6.8 (−16%)	<0.001
PVR (WU)	8.0 ± 1.2	6.0 ± 0.9	−2.0 (−25%)	<0.001
Fick CI (L/min/m^2^)	2.05 ± 0.13	2.15 ± 0.12	+0.10 (+5%)	0.04

Hemodynamic parameters measured by right heart catheterization (RHC) before initiation of inhaled treprostinil therapy and after approximately three months at maintenance dose. Values are presented as mean ± standard deviation (SD). Δ represents the mean absolute and percentage change from baseline. Abbreviations: mPAP = mean pulmonary artery pressure; PVR = pulmonary vascular resistance; CI = cardiac index; WU = Wood units.

## Data Availability

The data presented in this study are available on request from the corresponding author due to the data underlying this study contain protected health information (PHI) and cannot be shared publicly due to patient privacy and institutional restrictions. Deidentified data may be made available from the corresponding author upon reasonable request and with appropriate institutional approvals.

## Abbreviations

The following abbreviations are used in this manuscript:

AE	Adverse Event
CI	Cardiac Index
CPFE	Combined Pulmonary Fibrosis and Emphysema
CT	Computed Tomography
CTEPH	Chronic Thromboembolic Pulmonary Hypertension
DLCO	Diffusing Capacity for Carbon Monoxide
ERS	European Respiratory Society
ESC	European Society of Cardiology
FVC	Forced Vital Capacity
iTre	Inhaled Treprostinil
ILD	Interstitial Lung Disease
IPF	Idiopathic Pulmonary Fibrosis
IQR	Interquartile Range
MAP	Mean Arterial Pressure
mPAP	Mean Pulmonary Artery Pressure
NSIP	Nonspecific Interstitial Pneumonia
PAH	Pulmonary Arterial Hypertension
PAWP	Pulmonary Artery Wedge Pressure
PCWP	Pulmonary Capillary Wedge Pressure
PH	Pulmonary Hypertension
PH-ILD	Pulmonary Hypertension Associated with Interstitial Lung Disease
PVR	Pulmonary Vascular Resistance
QID	Four Times Daily
RAP	Right Atrial Pressure
RHC	Right Heart Catheterization
RT	Respiratory Therapist
RV	Right Ventricle
SD	Standard Deviation
SpO_2_	Peripheral Oxygen Saturation
WU	Wood Units
